# COVID-19 and unintended steps towards further equity in global health research

**DOI:** 10.1136/bmjgh-2023-011888

**Published:** 2023-06-16

**Authors:** Tamara Mulenga Willows, Jacquie Oliwa, Onesmus Onyango, Elibariki Mkumbo, John Maiba, Carl Otto Schell, Tim Baker, Jacob McKnight

**Affiliations:** 1 Tropical Medicine and Global Health, University of Oxford Medical Sciences Division, Oxford, UK; 2 Health Services Unit, KEMRI-Wellcome Trust Research Programme, Nairobi, Kenya; 3 Health Systems, Impact Evaluation and Policy, Ifakara Health Institute, Ifakara, Tanzania; 4 Department of Global Public Health, Karolinska Institute, Stockholm, Sweden; 5 Center for Tropical Medicine and Global Health, University of Oxford Centre for Tropical Medicine, Oxford, UK

**Keywords:** COVID-19, Health systems, Health services research, Hospital-based study, Qualitative study

## Abstract

There was, and possibly still is, potential for COVID-19 to disrupt power inequities and contribute to positive transformation in global health research that increases equity. While there is consensus about the need to decolonise by transforming global health, and a roadmap outlining how we could approach it, there are few examples of steps that could be taken to transform the mechanics of global health research. This paper contributes lessons learnt from experiences and reflections of our diverse multinational team of researchers involved in a multicountry research project. We demonstrate the positive impact on our research project of making further steps towards improving equity within our research practices. Some of the approaches adopted include redistributing power to researchers from the countries of interest at various stages in their career, by involving the whole team in decisions about the research; meaningfully involving the whole team in research data analysis; and providing opportunities for all researchers from the countries of interest to voice their perspectives as first authors in publications. Although this approach is consistent with how research guidance suggests research should be run, in reality it does not often happen in this way. The authors of this paper hope that by sharing our experience, we can contribute towards discussions about the processes required to continue developing a global health sector that is equitable and inclusive.

**Summary box**

- The colonial undertones in global health, underpinned by a history of side-lining people from low-income and middle-income countries from the design of global and public health systems, is a widely acknowledged problem.Decolonising global health academia, programming and financing, will entail a shift in leadership, power distribution and knowledge creation norms.
- The changes to global health practice brought about by COVID-19 provided opportunities to further decolonise research by redistributing some of the power around who shapes knowledge from mainly global north researchers to global south researchers at varying levels.
- We demonstrate the benefit of including researchers from the countries of interest with varying degrees of seniority in understanding ethical, practical and political dilemmas encountered during a multicountry research project.
- The way of working we evolved demonstrates an approach to increase equity in global health practice by decentralising decision-making and knowledge creation when conducting research. Providing the space for junior researchers to interpret data and shape the direction of research by developing academic papers are some examples of how this paper might affect practice.

## Background

The influence of colonial rule on global health and efforts to unpack is a growing area of concern for global health researchers. In Packard’s history of global health, he outlines how colonial rule and colonial medicine were inextricably entangled, setting the stage for a centralised, western biomedical worldview focused on short-term solutions.^1^ This same rationale would dictate decision-making and the focus of global health programmes and research even after the end of colonial rule. This culminated in magic bullet solutions, health interventions developed outside of the countries where the health problems exist, and little attention given to supporting the development of basic health services.^1^ However, as early as 1932, researchers recognised how a more decentralised approach to global health programmes would yield better long-term results.^1^

Atuire and Rutazibwa describe present day coloniality as a ‘way to engage colonialism in the present and anywhere (internally, bilaterally, globally) as a (re)production of extreme power inequality and the different institutions created to perpetuate this’,^2^ a description that characterises many sectors of global health today. They go further to describe decolonisation as ‘actively retrieving and cultivating agency in health and healthcare, including unearthing erased and delegitimised health systems’.^2^ Other contributors to this topic have further described decolonisation of global health as focused on digging out the deep roots of colonial structures and thinking which are not unique to global health in order to move towards an equitable and just world.^3–5^ Khan *et al* highlight how an arbitrary choice of interventions in both programmes and academic work or research topics, with little coordination or engagement with people on the receiving end, leads to top-down health programmes that cannot be sustained and perpetuate inequalities in communities.^6^

March 2020 marked the start of a seismic shift in how global health research, which we believe is intertwined with global health practice, would be carried out for the foreseeable future. In response to the risks posed by the COVID-19 pandemic, governments around the world introduced restrictions on movement, forcing many to adopt a new remote way of working. The pandemic also provided much for practitioners of global health to reflect on, such as which research projects are essential and how we protect those working in the sector. Senior practitioners in the sector outlined opportunities for change in a postpandemic world, with a particular focus on decolonising and decentralising global health as we ‘build back better’.^7–9^

The potential positive impact of making steps towards decolonising global health have been outlined with much greater vigour since the COVID-19 pandemic began, as this pandemic exposed the imbalances of power and risk that define the field.^10^ While there is a growing field of literature exploring the implications of COVID-19 for specific areas of research, little is known about how the COVID-19 pandemic has influenced changes in how global health researchers approach their work with respect to values, ways of working and priorities.^11 12^ One such implication is a departure from rigorous methodology such as in participatory research where methods such as social network analysis, typically done face to face, were done conducted online without a prior assessment of this would affect the results and interaction with participants.^11^

In response to Abimbola *et al* and other researchers’ calls to decentralise and decolonise global health, we wish to reflect on how COVID-19 did indeed offer opportunities to alter our practices and to share some ways in which our work during the Wellcome Trust funded POETIC (Provision of Essential Treatment in Critical Care in COVID-19) project allowed us to take further steps towards decolonising our research practices. Our experience as a multinational research team is particularly novel in that we worked across two high-income countries (HICs) (Sweden and the UK) and two lower-income and middle-income countries (LMICs) (Kenya and Tanzania) with strongly contrasting approaches to managing the pandemic.

In this paper, we, as authors, reflect on our experience during the POETIC project outlined in box 1. First, we will discuss how POETIC’s geographically dispersed teams and remote working approach forced us to partially reconfigure traditional power dynamics common in global health partnerships. Second, we will explore ethics in practice for the POETIC project and how the POETIC team navigated the situated ethics questions that emerged. The authors of this paper hope that by sharing our experience, we can further the process of developing a global health sector that is fair and truly representative.

Box 1 **A brief outline of the Provision of Essential Treatment in Critical Care in COVID-19 (POETIC) project and how it was intended to work^45–47^**

**POETIC project**

The POETIC project is a Wellcome Trust funded ‘collaboration between institutions in Kenya, Tanzania, UK and Sweden to investigate critical care approaches including consensus generation, health economic analysis, health facility assessments, surveys and in-depth interviews of front-line health-workers and stakeholders, with the goal of output relevant to policy and improving critical care’.

The project focuses on investigating critical illness care in various hospitals in Kenya and Tanzania to understand the benefits that might be experienced if essential emergency and critical care was prioritised for all critically ill patients in hospitals in the two countries.

## Who are the POETIC project team?

The POETIC project team, created in 2020, consists of staff at five academic and research organisations: Ifakara Health Institute (IHI) (Tanzania); Kenya Medical Research Institute (KEMRI) Wellcome Trust Research Programme (KWTRP); University of Oxford Health Systems Collaborative (HSC) (UK); Uppsala University (Sweden) and the London School of Hygiene and Tropical Medicine (UK). We accept that KWTRP and IHI are well-established organisation with comparatively more power and influence in the health research space within their respective countries than less well-established organisations in Kenya and Tanzania. Therefore, staff at these organisations would have relatively more power to negotiate the dynamics of their relationship with research partners at institutions in high-resource settings. The first author of this paper is a dual Zambian and British citizen who has spent an equal amount of time living and working in both countries, while many of the authors have varied experiences living and working between Europe and East Africa. The principal investigators of POETIC are affiliated with Swedish, UK and Tanzanian organisations and based in Sweden and Tanzania. The junior researchers involved in POETIC, as well as this paper, are Kenyan and Tanzanian researchers based in their respective countries, while senior researchers included a mix of British and Kenyan researchers who either live in or have lived experience in East Africa.

After meeting in-person for the first time as a team, nearly 2 years after our POETIC began, we were able to create the space for informal conversations about how the COVID-19 pandemic affected the way we worked during this study. Many of us had not worked together before so were unaware of each other’s thoughts on or efforts to make working in global health research more equitable or inclusive. However, the experiences we describe will make us even more intentional about embedding equity and inclusion in all aspects of our global health practice irrespective of whether travel becomes more widespread after the pandemic.

## Decentralising power

To address the first aim of this paper, we will first explore one key challenge: reconfiguring team hierarchy. This is often discussed when the subjects are health managers and health workers, but not global health researchers.^13–16^ We looked to literature from the business world, in addition to global health fields such as health systems, because business literature includes a much broader discussion on decentralisation and distributed leadership among researchers rather than only participants of research.^17–21^ Before the COVID-19 era, there was an established yet unspoken chain of command in much of global health research: directives issued from HICs were enacted by LMIC partners with little input sought from enacting partners.^22^ Eichbaum *et al* and Khan *et al* describe how, often, an HIC representative would be flown in to lead a project to purportedly offer technical expertise and build capacity.^6 23^

Within organisational studies literature, teams that try to maintain traditional team hierarchy while working remotely are thought to struggle for a number of reasons. These include infrequent communication from team leaders when working virtually and the absence of a team leader within the same physical spaces as their team members. These changes, brought about by remote working, result in a loss of the associated positive psychological impact a leader’s physical presence.^13^ Working remotely across teams became the norm for the POETIC project due to COVID-19 and some of the team members in the UK charged with leading the qualitative work recognised that Swedish and British researchers’ inability to visit the projects meant that we could not follow the standard model. Hence, we decentralised some aspects of the research to colleagues of all levels in Kenya and Tanzania which was not only more equitable, but also more practical. Although the overall leadership of our project was HIC dominant, although not exclusively so, this change allowed us distribute some of the power to more members of the team based in Kenya and Tanzania. Prior to our project, this approach was already being embraced and prioritised by many of the institutions involved in this paper as illustrated through programmes such as KWTRP’s Initiative to Develop African Research Leaders (IDeAL) programme.^24 25^ Additionally, authors in the decolonisation space^26^ have also advocated for resituating decision-making to partners outside of HICs in global health projects, but this process of resituating decision-making and project design was pushed forward by pandemic necessities. There are numerous examples within global health of distributed leadership models among healthcare managers and healthcare workers, but papers on this topic focusing on global health researchers are scarce.^20 21^

For POETIC, the pandemic forced our team to rely largely on Microsoft Teams and Zoom for communication in our project. The physical distance between Kenyan and Tanzanian researchers, and those with in the UK and Sweden with supervisory roles, resulted in the latter greatly relying on in-country teams.

It is important to state here that within our team, we had different ideas of how the research work would be carried out based on our previous experiences. Some members envisioned less involvement of Kenyan and Tanzanian junior researchers at the analysis stage as is described in other studies in the literature, and others expected that the research would be led by Kenyan and Tanzanian researchers of differing levels of seniority.^27^ Researchers based in the UK relied on Kenyan and Tanzanian counterparts not only to collect data but also to play a central role in framing results through biweekly ‘data reflection’ meetings, which we used for reporting, discussing findings and setting new priorities. In these meetings, Swedish and British researchers were forced to acknowledge their unfamiliarity with the context and as such, it allowed more room for Kenyan and Tanzanian researchers to express themselves and their ideas about what the data revealed the state of critical illness care delivery. Additionally, Kenyan and Tanzanian researchers provided contextually relevant information about the data collected. One example of this is when they clarified that if participants in interviewers mentioned ‘different motivations and behaviours’, they were referring to money influencing how much additional work some hospital staff engage in. They were able to confirm this with the participants who felt comfortable disclosing this information to them off the record. Prior to POETIC, some of the junior researchers in Tanzania and Kenya had not been invited to participate in data analysis after collecting data and if they were, these were always led by senior researchers who had not participated in data collection. Equally, some of the UK-based team members had only conducted data reflection meetings with other senior researchers and had not involved those who collected the data in analysing it. While this process benefited researchers in Kenya and Tanzania, we acknowledge that those in the UK and Sweden had to become comfortable with ceding control of the decision-making process. This process of acceptance was made easier because of the results produced through this process such as the rich analysis of qualitative data led by Kenyan and Tanzanian junior researchers. A further area of positive change was in ensuring that all of the team participated in academic writing including junior Kenyan and Tanzanian researchers leading on papers as first authors.^28^ This practice was adopted by other studies within our institutions before the pandemic but is not commonplace in all research institutions.^27 29^

We also held data sense checking meetings once transcripts had been received and read by researchers in Kenya, Tanzanian and the UK: we define these as semistructured meetings where a topic from transcripts were chosen and discussed by research assistants in Kenya and Tanzania. These meetings allowed for data collectors to provide more context for findings to the rest of the team as lockdown restricted the HIC-based members of the team from visiting facilities to understand the context of where data were collected. They also acted as an additional way to verify our data. It is uncommon within global health for junior researchers, who collect data, to also analyse and disseminate findings.^30^ It is also uncommon for research participants from resource abundant and resource constrained health settings to shape and provide their perspective on findings before they are finalised.^31 32^ In this case, researchers in the UK and Sweden could not justifiably interpret the findings, particularly the qualitative findings, on their own without setting foot in either country, therefore, diminishing the typical authority researchers in HICs have within global health studies and in some unintended way, decolonising our research process. We all recognise the issue pointed out by Khan *et al*: although we had notionally supported decolonisation as individuals prior to the pandemic, we had not all had the exposure to how something might be done differently until the pandemic forced us to rethink some aspects of our research processes.^6^ This project provided a real opportunity to reflect on how initial changes to the power dynamics within our work can bring us closer to decolonising our practice.

Lawrence and Hirsch challenge us further by highlighting that only a small number of partnerships have responded to calls for research collaborations to involve researchers from the countries where studies are conducted at all levels of the research process, including publication and grant writing.^33^ Our team’s experience of working virtually caused those of us in the UK and Sweden to rely heavily on the rest of the team working in Kenya and Tanzania to shape POETIC’s research findings, providing space for their voices in knowledge creation.

## Ethical considerations

While COVID-19 led the POETIC team to redress some injustices experienced when creating and determining what counts as global health knowledge,^34^ the POETIC team were unable to adequately address certain ethical issues raised by the pandemic. While the organisations that support us do not contend to have found the solution to all ethical struggles, we attempt to present some of the ethical struggles we experienced within existing thoughtful ethical considerations. By November 2020, when our data collection was in full swing, Kenya was experiencing approximately 100 official COVID-19 deaths per week.^35^ The situation in Tanzania was less clear due to a limited dissemination of information.^36^ With these risks in mind, there were two questions the POETIC management team (which included senior researchers from all the countries involved) had to grapple with: (1) How necessary was it to conduct this project during the pandemic and (2) How could participants and the project team be protected from the risks associated with continuing our project?

The decision to continue or postpone research studies has been something many research institutions have had to consider during this pandemic.^37^ For POETIC, this decision required little debate as the process each institution has in place to review project proposals before they are permitted is trusted by all team members and the findings from this project are intended to increase the survival of patients that become critically unwell, including from COVID-19.^38^ Ethical review boards in Kenya, Tanzania and the UK agreed and we were granted permission to begin. However, the project proposal did not outline explore the ethics of which researchers would be exposed to COVID-19 while undertaking project work beyond assessing risks of contracting COVID-19 and environmental safety assessment, and the underlying power dynamics involved in that decision. While research assistants in Kenya and Tanzania within our team did have an opportunity before the study to discuss which risks they were being exposed to during the study, we recognise this does not happen often enough across studies at other institutions.

POETIC’s leadership, primarily from HICs, factored in training on personal protective equipment (PPE) and how to use it for optimum protection. However, specific preparation for the death and distress our researchers would witness was not formally included in our project protocol or training although we attempted to address this after we began our project when a death occurred. Historically, ethical considerations for research studies during pandemics have been overlooked.^39^ While we believe receiving reviews from multiple ethical review boards was valuable for providing perspectives from Kenya, Tanzania and the UK, they perhaps did not fully explore all the ethical challenges of producing rigorous data under high-risk conditions. Notably exposure to a contagious, potentially fatal disease such as COVID-19 and the psychological impact of working in health facilities during a deadly pandemic which would be present regardless of who is leading a study in this environment.^40^ Additionally, the power dynamics between researchers from influential high-income institutions and those from less academically influential institutions in-country within global health projects mean questions about risk are less likely to come from teams based and living in countries where studies take place. This is because researchers from institutions that are perceived to have less power in a research partnership are unlikely to highlight these risks to ensure their contracts are renewed.^41^

In POETIC, we partially addressed this ethical quandary because the greater level of involvement of researchers of all levels from Kenya and Tanzania, meant those researchers felt more comfortable proposing changes to POETIC’s research schedule after receiving ethical approval. This in-turn helped junior researchers from Kenya and Tanzania decrease their risk of contracting COVID-19 by highlighting to the team when it would be safe to collect data and how best to go about doing so. This greater level of involvement also meant Kenyan and Tanzanian team members felt comfortable discussing psychologically distressing experiences with team members from HICs after the project began. When Kenyan and Tanzanian researchers had the opportunity to take up more decision-making power during POETIC, it helped to create an environment where ethical issues not previously considered could be addressed. It is this acknowledgement of power dynamics, even in ethical considerations and taking seemingly preliminary steps to address them, that demonstrate how studies can work towards decolonising global health practice. Matters of ethics in practice are difficult to anticipate, even when protocols are reviewed by the most thorough ethical review boards.^42 43^ We must be sensitive to the range of risks we expose our colleagues to, make efforts to minimise them and ensure appropriate compensation and recognition through publications are provided. While partnerships that bring together professionals from around the world have their advantages, the asymmetry of power and the associated risk to researchers based in a country where a study takes place should be re-examined in a post-COVID-19 era of global health.^44^

To further convey the experiences of junior researchers from Kenya and Tanzania during POETIC, we have included excerpts from a focused conversation with them on this topic in box 2.

Box 2 **Excerpts from a small group discussion with Provision of Essential Treatment in Critical Care in COVID-19 (POETIC) Kenyan and Tanzanian Research assistants led by the first author exploring some topics outlined in this paper. These discussions demonstrate the benefits and challenges of altering our research practices for junior researchers in Kenya and Tanzania.**

**Edited group discussion transcription**

**On working within geographically dispersed teams**

Research assistant 1: It [working remotely] was something that we felt was necessary, but I wasn’t so sure things would go well. But you just need to plan well, and I now feel I know the people behind the screens. We have shown great cohesion and teamwork even before we meet in person which has been a great experience. I had an online induction and didn’t have a baseline for how to engage with the team but [my supervisor] made [themselves] available not only through email but other means so I could contact [them] easily at any time. COVID-19 programmed us to adapt to working virtually and running things without having team member’s physically present. I didn’t have a baseline to compare what working in person with this team would be. The support I got from my supervisor has been so good I didn’t miss in-person supervision.

Research assistant 2: Nothing is equal to a physical meeting, but I feel we have gained a lot through the model we have used. If we had the option of meeting in person maybe, we wouldn’t have been in contact as frequently as we have been. I also feel technology has helped increase the frequency of us contacting each other but it might not be the same as physical contact. Its not only about the frequency but the quality of our frequent contacts. Sometimes when you have these calls, the way the agenda and contributions are set can make you feel even more distant from the team.

Research assistant 3: Being in the same physical space can be very useful especially when you want to ask someone something you don’t need to make a lot of arrangements like we do when we need to call those [team members] that are far away [outside of Tanzania]. Being in the same physical space can help with improving team working as well.

**On the practicalities of working during COVID-19**

Research assistant 1: Politically, sometimes you get to a hospital where things are too political, so people have this idea that any research work that comes in, people (local researchers) have ‘eaten’ (received additional money). So somebody has ‘eaten’ somewhere. Yeah. So everybody wants to have a bite, and people would always think about it like that. You get it. So when you are this very junior researcher on the ground, sometimes you get to a point where you are pushed and you will feel like, ‘Well, this is way above me [giving out money]’, but I think we’ve always had that help.…From the ground level, there’s that contextual kind of advice or view that it’s only obtained when you have the supervisor or the team leads on the ground influencing how this work is done.

Research assistant 2: When we started the data reflection meetings, I was sceptical about it working but the conversation flowed. However, I don’t think the information flowed as well to the team leaders even though we shared what was happening. This is a lesson for us to find a better way to bring together information for those who operate in different ways that is, how info is received by those focused on qual work vs quant work. I think more detail should have been placed in the protocol to reduce this confusion.

We have summarised the experiences discussed in this paper and the steps we took to achieve them in table 1 to aid others who wish to make similar changes to their research practice.

**Table 1:** An outline of our recommended actions for more equitable research practice and the steps we took to make those changes

Recommendations for more equitable research practice	Steps we took to achieve this
Create a cohesive team environment	Step 1. Research assistants in Kenya, the UK and Tanzania undertook online training individually before starting the project. Step 2. To help junior researchers across all sites feel more connected to the team while working online, both their direct supervisors and the Project PIs met with them via video call to introduce themselves and provided various mediums they could use to contact them. Step 3. PIs and direct supervisors in Kenya, Tanzania and the UK made themselves available often to support junior team members virtually in adjusting to remotely working on this study.
Create opportunities for all researchers to participate in research administration decisions	Step 1. Kenyan and Tanzanian research assistants were asked by research leadership in Kenya, Sweden, Tanzania and the UK to make suggestions about the most appropriate time to collect data and the mode of data collection (in-person vs video call vs phone call). Step 2. Kenyan and Tanzanian researchers were invited to share thoughts on risks they faced to their physical safety from COVID-19 at general weekly team meetings. Step 3. During weekly data reflection meetings with British and Kenyan senior researchers, Kenyan and Tanzanian research assistants were invited to share reflections about witnessing deaths of patients in the hospitals during data collection and ways the rest of the team could support them.
Create opportunities for inclusive knowledge creation	Step 1. Senior researchers in the UK proposed weekly data reflection meetings where junior researchers in Kenya and Tanzania who had collected the data could lead discussions on what the data revealed. Step 2. Senior researchers in Kenya, the UK, Sweden and Tanzania reviewed data analysis presentations created by junior researchers across all the sites and supported them in presenting our findings to participants. Step 3. Participants at all study sites were offered the opportunity to hear these presentations and provide feedback on findings before they were finalised during data sense checking meetings. Step 4. All junior researchers in POETIC were invited to propose ideas for peer-reviewed papers based on the data analysed and supported by senior researchers to submit these papers for publication as first authors. Step 5. All junior researchers were offered opportunities to present the study findings to external stakeholders in Kenya, Tanzania and internationally.

PIs, principal investigators; POETIC, Provision of Essential Treatment in Critical Care in COVID-19.

## Conclusion

The COVID-19 pandemic has exposed ethical, logistical and political dilemmas for global health research. We believe these issues were always there but became more salient as the pandemic took hold. Addressing some of these dilemmas through a decolonisation lens could help global health practitioners build a more inclusive sector where voices that were previously not given enough opportunity in the literature, are afforded the same opportunities as colleagues in HICs. We believe there are some lessons to be learnt from aspects of organisational studies which have a longer history of working virtually and exploring the impact of that on power distribution within teams. While the POETIC project does not attempt to position itself as the model for ideal decolonised global health practice, our experience demonstrates how COVID-19 provided opportunities to reflect on existing practice within our institutions and continue to explore ways of working that give greater authority to the voices of a wide range of researchers from the countries where the work is conducted. Admittedly, the steps we describe towards further decolonising our practice were partial and not initially intended by all members of the team. Decolonisation will require many more fundamental structural shifts and challenging conversations between and within research teams, considerations about what will be lost and gained for individuals and academic institutions, and most notably funding bodies. Despite this, we believe COVID-19 has shown us that if all researchers both in resource abundant and resource limited settings are willing to be led by those embedded in the contexts we are trying to affect, it might not be hard to be better.

## Data Availability

No data are available. Not applicable.
